# Relaxed alternating CQ algorithms for the split equality problem in Hilbert spaces

**DOI:** 10.1186/s13660-018-1933-2

**Published:** 2018-12-07

**Authors:** Hai Yu, Fenghui Wang

**Affiliations:** Department of Mathematics, Luoyang Normal University, Luoyang, P.R. China

**Keywords:** 47J25, 47J20, 49N45, 65J15, Split feasibility problem, Split equality problem, Relaxed alternating method

## Abstract

In this paper, we are concerned with the split equality problem (SEP) in Hilbert spaces. By converting it to a coupled fixed-point equation, we propose a new algorithm for solving the SEP. Whenever the convex sets involved are level sets of given convex functionals, we propose two new relaxed alternating algorithms for the SEP. The first relaxed algorithm is shown to be weakly convergent and the second strongly convergent. A new idea is introduced in order to prove strong convergence of the second relaxed algorithm, which gives an affirmative answer to Moudafi’s question. Finally, preliminary numerical results show the efficiency of the proposed algorithms.

## Introduction

The split feasibility problem (SFP) was first introduced by Censor and Elfving [5]. It models various inverse problems arising from phase retrievals and medical image reconstruction [3]. More specifically, the SFP requires to find a point $x\in H_{1}$ satisfying the property

1.1 $$ x\in C \quad \text{and}\quad Ax\in Q, $$

where *C* and *Q* are nonempty, closed and convex subsets of real Hilbert spaces $H_{1}$ and $H_{2}$, respectively, and $A: H_{1}\rightarrow H_{2}$ is a bounded linear operator.

Various iterative methods have been constructed to solve the SFP (1.1); see [3–5, 16, 19, 20, 22–25, 28]. One of the well-known methods appearing in the literature for solving the SFP is Byrne’s CQ algorithm [3, 4], which generates a sequence $\{x_{n}\}$ by the recursive procedure

1.2 $$ x_{n+1}=P_{C}\bigl(x_{n}-\gamma A^{*}(I-P_{Q})Ax_{n}\bigr), $$

where $\gamma\in(0,\frac{2}{\|A\|^{2}})$, $P_{C}$ and $P_{Q}$ are projections onto *C* and *Q*, respectively, *I* denotes the identity operator, and $A^{*}$ denotes the adjoint of *A*. The SFP can be also solved by a different method [17, 27], namely

1.3 $$ x_{n+1}=x_{n}-\gamma\bigl[(I-P_{C})x_{n}+A^{*}(I-P_{Q})Ax_{n} \bigr], $$

where *γ* is a properly chosen parameter. In Hilbert spaces, both (1.2) and (1.3) converge weakly to a solution of the SFP whenever such a solution exists.

Recently, Moudafi [11] introduced the split equality problem (SEP):

1.4 $$ \text{Find}\quad x\in C, y\in Q \quad \text{such that}\quad Ax=By, $$

where $H_{1}$, $H_{2}$, $H_{3}$ are real Hilbert spaces, $C\subseteq H_{1}$, $Q\subseteq H_{2}$ are two nonempty, closed and convex subsets, and $A: H_{1}\rightarrow H_{3}$, $B: H_{2}\rightarrow H_{3}$ are two bounded linear operators. It is clear that the SEP is more general than the SFP. As a matter of fact, if $B=I$ and $H_{3}=H_{2}$, then the SEP (1.4) reduces to the SFP (1.1). Algorithms for solving the SEP have received great attention; see, for instance, [6, 7, 10–12, 14, 18]. Among these works, Moudafi [11] introduced the alternating CQ-algorithm (ACQA), namely

1.5 $$ \textstyle\begin{cases} x_{n+1}=P_{C}(x_{n}-\gamma_{n}A^{*}(Ax_{n}-By_{n})), \\ y_{n+1}=P_{Q}(y_{n}+\gamma_{n}B^{*}(Ax_{n+1}-By_{n})). \end{cases} $$

It is shown that the sequence $\{(x_{n}, y_{n})\}$ produced by ACQA converges weakly to a solution of (1.4) provided that the solution set $S=\{(x, y)\in C \times Q \mid Ax=By\}$ is nonempty and $\{\gamma_{n}\}$ is a positive nondecreasing sequence such that $\gamma_{n}\in (\epsilon, \min(\frac{1}{\|A\|^{2}}, \frac{1}{\|B\|^{2}})-\epsilon )$ for a small enough $\epsilon>0$.

However, the ACQA might be hard to implement whenever $P_{C}$ or $P_{Q}$ fails to have a closed-form expression. A typical example of such a situation is the level set of convex functions. Indeed, Moudafi [10] considered the case when *C* and *Q* are level sets:

1.6 $$ C=\bigl\{ x\in H_{1} \mid c(x)\leq0\bigr\} $$

and

1.7 $$ Q=\bigl\{ y\in H_{2} \mid q(y)\leq0\bigr\} , $$

where $c:H_{1}\rightarrow\mathbb{R}$ and $q:H_{2}\rightarrow \mathbb{R}$ are two convex and subdifferentiable functions on $H_{1}$ and $H_{2}$, respectively. Here the subdifferential operators *∂c* and *∂q* of *c* and *q* are assumed to be bounded, i.e., bounded on bounded sets. In this case, it is known that the associated projections are very hard to calculate. To overcome this difficulty, Moudafi [10] presented the relaxed alternating CQ-algorithm (RACQA):

1.8 $$ \textstyle\begin{cases} x_{n+1}=P_{C_{n}}(x_{n}-\gamma A^{*}(Ax_{n}-By_{n})), \\ y_{n+1}=P_{Q_{n}}(y_{n}+\gamma B^{*}(Ax_{n+1}-By_{n})). \end{cases} $$

where $\gamma\in (0, \min(\frac{1}{\|A\|^{2}}, \frac{1}{\|B\|^{2}}) )$, $\{C_{n}\}$ and $\{Q_{n}\}$ are two sequences of closed convex sets defined by

1.9 $$ C_{n}=\bigl\{ x\in H_{1} \mid c(x_{n})+\langle\xi_{n}, x-x_{n}\rangle\leq0 \bigr\} ,\quad \xi_{n}\in\partial c(x_{n}), $$

and

1.10 $$ Q_{n}=\bigl\{ y\in H_{2} \mid q(y_{n})+\langle\eta_{n}, y-y_{n}\rangle\leq0 \bigr\} ,\quad \eta_{n}\in\partial q(y_{n}). $$

Since $C_{n}$ and $Q_{n}$ are clearly half-spaces, the associated projections thus have closed form expressions. This indicates that the implementation of RACQA is very easy. Under suitable conditions, Moudafi [10] proved that the sequence $\{(x_{n}, y_{n})\}$ generated by the RACQA converges weakly to a solution of (1.4). Meanwhile, he raised the following open question in [10].

### Question 1.1

Is there any strong convergence theorem of an alternating algorithm for the SEP (1.4) in real Hilbert spaces?

Motivated by the works mentioned above, we continue to study the SEP. We will treat the SEP in a different way. Indeed, we will prove that the SEP amounts to solving the coupled fixed point equation:

1.11 $$ \textstyle\begin{cases} x=x-\tau[(x-P_{C}x)+A^{*}(Ax-By)], \\ y=y-\tau[(y-P_{Q}y)-B^{*}(Ax-By)], \end{cases} $$

where *τ* is a positive real number. This equation enables us to propose a new algorithm for solving the SEP. We also consider the case when the convex sets involved are level sets of given convex functionals. Inspired by (1.11) and the relaxed projection algorithm, we propose two new relaxed alternating algorithms for the SEP governed by level sets, which present an affirmative answer to Moudafi’s question. Finally, we give numerical results for the split equality problem to demonstrate the feasibility and efficiency of the proposed algorithms.

## Preliminaries

Throughout this paper, we always assume that *H* is a real Hilbert space with the inner product $\langle\cdot, \cdot\rangle$ and norm $\|\cdot\|$. We denote by *I* the identity operator on *H*, and by $\operatorname{Fix}(T)$ the set of the fixed points of an operator *T*. The notation → stands for strong convergence and ⇀ stands for weak convergence.

### Definition 2.1

([2, 4])

Let $T:H\rightarrow H$ be an operator. Then *T* is

1. nonexpansive if
  $$\|Tx-Ty\| \leq\|x-y\|,\quad \forall x, y\in H; $$
2. firmly nonexpansive if
  $$\Vert Tx-Ty \Vert ^{2}\leq \Vert x-y \Vert ^{2}- \bigl\Vert (I-T)x-(I-T)y \bigr\Vert ^{2},\quad \forall x,y\in H. $$

Let *C* be a nonempty, closed and convex subset of *H*. For any $x\in H$, the projection onto *C* is defined as

$$P_{C} x= \operatorname{argmin}\bigl\{ \Vert y-x \Vert \mid y\in C \bigr\} . $$

The projection $P_{C}$ has the following well-known properties.

### Lemma 2.2

([2, 15])

*For all* $x,y\in H$,

1. $\langle x-P_{C} x, z-P_{C} x\rangle\leq0$, $\forall z\in C$;
2. $P_{C}$ *is nonexpansive*;
3. $P_{C}$ *is firmly nonexpansive*;
4. $\langle P_{C}x-P_{C}y, x-y\rangle\geq\|P_{C}x-P_{C}y\|^{2}$.

### Definition 2.3

Let $T: H\rightarrow H$ be an operator with $\operatorname{Fix}(T)\neq \emptyset$. Then $I-T$ is said to be demiclosed at zero, if, for any $\{x_{n}\}$ in *H*, the following implication holds:

$$ x_{n}\rightharpoonup x \quad \text{and}\quad (I-T)x_{n} \rightarrow0 \quad \Rightarrow \quad x\in\operatorname{Fix}(T). $$

It is well known that if *T* is a nonexpansive operator, then $I-T$ is demiclosed at zero. Since the projection $P_{C}$ is nonexpansive, then $I-P_{C}$ is demiclosed at zero.

### Definition 2.4

Let $\lambda\in(0,1)$ and $f: H\rightarrow(-\infty, +\infty]$ be a proper function.

1. *f* is convex if
  $$f\bigl(\lambda x+(1-\lambda)y\bigr)\leq\lambda f(x)+(1-\lambda)f(y),\quad \forall x,y\in H. $$
2. A vector $u\in H$ is a subgradient of *f* at a point *x* if
  $$f(y)\geq f(x)+\langle u, y-x\rangle,\quad \forall y\in H. $$
3. The set of all subgradients of *f* at *x*, denoted by $\partial f(x)$, is called the subdifferential of *f*.

To prove our main results, we need the following lemmas.

### Lemma 2.5

*For all* $x, y\in H$, *we have*

$$\|x+y\|^{2}\leq\|y\|^{2}+2\langle x, x+y\rangle. $$

### Lemma 2.6

([21])

*Let* $\{a_{n}\}$ *be a sequence of nonnegative real numbers such that*

$$ a_{n+1}\leq(1-\gamma_{n})a_{n}+ \gamma_{n} \delta_{n},\quad n\geq0, $$

*where* $\{\gamma_{n}\}$ *is a sequence in* $(0, 1)$ *and* $\{\delta_{n}\}$ *is a sequence in* $\mathbb{R}$ *such that*

1. $\sum_{n=0}^{\infty}\gamma_{n}=\infty$;
2. $\limsup_{n\rightarrow\infty}\delta_{n}\leq0$ *or* $\sum_{n=0}^{\infty}|\delta_{n}\gamma_{n}|<\infty$.

*Then*, $\lim_{n\rightarrow\infty}a_{n}=0$.

## A new alternating CQ-algorithm

In what follows, we always assume that the solution set of the SEP is nonempty, i.e., $S=\{(x, y)\in C \times Q \mid Ax=By\}\neq\emptyset$. In order to solve problem (1.4), we need the following lemma, which has as a key role in later developments.

### Lemma 3.1

*An element* $(x, y)\in H_{1}\times H_{2}$ *solves* (1.4) *if and only if it solves the fixed point equation* (1.11).

### Proof

If $(x, y)$ solves (1.4), then $x=P_{C}x$, $y=P_{Q}y$ and $Ax=By$. It is obvious that the fixed point equation (1.11) holds.

To see the converse, let $(x, y)$ be a solution of equation (1.11). Then,

3.1 $$ \textstyle\begin{cases} (x-P_{C}x)+A^{*}(Ax-By)=0, \\ (y-P_{Q}y)-B^{*}(Ax-By)=0. \end{cases} $$

Choosing any $(\tilde{x}, \tilde{y})\in S$, we get

$$\begin{aligned} 0&=\bigl\langle (x-P_{C}x)+A^{*}(Ax-By), x-\tilde{x}\bigr\rangle \\ &=\langle x-P_{C}x, x-\tilde{x}\rangle+\langle Ax-By, Ax-A\tilde{x} \rangle, \end{aligned}$$

and

$$\begin{aligned} 0&=\bigl\langle (y-P_{Q}y)-B^{*}(Ax-By), y-\tilde{y}\bigr\rangle \\ &=\langle y-P_{Q}y, y-\tilde{y}\rangle-\langle Ax-By, By-B\tilde{y} \rangle. \end{aligned}$$

Adding the above two equalities, we have

$$\begin{aligned} 0&=\langle x-P_{C}x, x-\tilde{x}\rangle+\langle y-P_{Q}y, y-\tilde{y}\rangle+\|Ax-By\|^{2} \\ &=\langle x-P_{C}x, x-P_{C}x\rangle+\langle x-P_{C}x, P_{C}x-\tilde{x}\rangle+\langle y-P_{Q}y, y-P_{Q}y\rangle \\ &\quad {} +\langle y-P_{Q}y, P_{Q}y-\tilde{y}\rangle+ \|Ax-By\|^{2} \\ &\geq\|x-P_{C}x\|^{2}+\|y-P_{Q}y \|^{2}+\|Ax-By\|^{2}. \end{aligned}$$

Thus, $x=P_{C}x$, $y=P_{Q}y$ and $Ax=By$. That is, $(x, y)$ solves (1.4), and the proof is complete. □

Applying Lemma 3.1, we introduce a new alternating CQ-algorithm for the SEP (1.4).

### Algorithm 3.2

Let $(x_{0}, y_{0})\in H_{1}\times H_{2}$ be arbitrary. Given $(x_{n}, y_{n})$, construct $(x_{n+1}, y_{n+1})$ via the formula

3.2 $$ \textstyle\begin{cases} x_{n+1}=x_{n}-\tau[(x_{n}-P_{C}x_{n})+A^{*}(Ax_{n}-By_{n})], \\ y_{n+1}=y_{n}-\tau[(y_{n}-P_{Q}y_{n})-B^{*}(Ax_{n+1}-By_{n})], \end{cases} $$

where $0<\tau<(1+c)^{-1}$ with $c=\max(\|A\|^{2}, \|B\|^{2})$.

### Theorem 3.3

*Let* $\{(x_{n}, y_{n})\}$ *be the sequence generated by Algorithm* 3.2. *Then* $\{(x_{n}, y_{n})\}$ *converges weakly to a solution of the SEP* (1.4).

### Proof

Let $(x^{*}, y^{*})\in S$. Then $x^{*}\in C$, $y^{*}\in Q$ and $Ax^{*}=By^{*}$. In view of (3.2), Lemma 2.2 and Young’s inequality, we conclude that

$$\begin{aligned} \bigl\Vert x_{n+1}-x^{*} \bigr\Vert ^{2}&= \bigl\Vert x_{n}-x^{*} \bigr\Vert ^{2}-2\tau\bigl\langle (x_{n}-P_{C}x_{n})+A^{*}(Ax_{n}-By_{n}), x_{n}-x^{*}\bigr\rangle \\ &\quad {} +\tau^{2} \bigl\Vert (x_{n}-P_{C}x_{n})+A^{*}(Ax_{n}-By_{n}) \bigr\Vert ^{2} \\ &\leq \bigl\Vert x_{n}-x^{*} \bigr\Vert ^{2}-2\tau\bigl\langle x_{n}-P_{C}x_{n}, x_{n}-x^{*} \bigr\rangle -2\tau\bigl\langle Ax_{n}-By_{n}, Ax_{n}-Ax^{*}\bigr\rangle \\ &\quad {} +\tau^{2} \biggl(\bigl(1+ \Vert A \Vert ^{2} \bigr) \Vert x_{n}-P_{C}x_{n} \Vert ^{2}+\biggl(1+\frac{1}{ \Vert A \Vert ^{2}}\biggr) \bigl\Vert A^{*}(Ax_{n}-By_{n}) \bigr\Vert ^{2} \biggr) \\ &\leq \bigl\Vert x_{n}-x^{*} \bigr\Vert ^{2}-2\tau \Vert x_{n}-P_{C}x_{n} \Vert ^{2}-2 \tau\bigl\langle Ax_{n}-By_{n}, Ax_{n}-Ax^{*}\bigr\rangle \\ &\quad {} +\tau^{2}\bigl(1+ \Vert A \Vert ^{2}\bigr) \bigl( \Vert x_{n}-P_{C}x_{n} \Vert ^{2}+ \Vert Ax_{n}-By_{n} \Vert ^{2}\bigr). \end{aligned}$$

Similarly, we obtain

$$\begin{aligned} \bigl\Vert y_{n+1}-y^{*} \bigr\Vert ^{2} &= \bigl\Vert y_{n}-y^{*} \bigr\Vert ^{2}-2\tau\bigl\langle (y_{n}-P_{Q}y_{n})-B^{*}(Ax_{n+1}-By_{n}), y_{n}-y^{*}\bigr\rangle \\ &\quad {} +\tau^{2} \bigl\Vert (y_{n}-P_{Q}y_{n})-B^{*}(Ax_{n+1}-By_{n}) \bigr\Vert ^{2} \\ &\leq \bigl\Vert y_{n}-y^{*} \bigr\Vert ^{2}-2\tau\bigl\langle y_{n}-P_{Q}y_{n}, y_{n}-y^{*} \bigr\rangle +2\tau\bigl\langle Ax_{n+1}-By_{n}, By_{n}-By^{*}\bigr\rangle \\ &\quad {} +\tau^{2} \biggl(\bigl(1+ \Vert B \Vert ^{2} \bigr) \Vert y_{n}-P_{Q}y_{n} \Vert ^{2}+\biggl(1+\frac{1}{ \Vert B \Vert ^{2}}\biggr) \bigl\Vert B^{*}(Ax_{n+1}-By_{n}) \bigr\Vert ^{2} \biggr) \\ &\leq \bigl\Vert y_{n}-y^{*} \bigr\Vert ^{2}-2\tau \Vert y_{n}-P_{Q}y_{n} \Vert ^{2}+2 \tau\bigl\langle Ax_{n+1}-By_{n}, By_{n}-By^{*}\bigr\rangle \\ &\quad {} +\tau^{2}\bigl(1+ \Vert B \Vert ^{2}\bigr) \bigl( \Vert y_{n}-P_{Q}y_{n} \Vert ^{2}+ \Vert Ax_{n+1}-By_{n} \Vert ^{2}\bigr). \end{aligned}$$

On the other hand, we have

$$\begin{aligned} & 2\bigl\langle Ax_{n}-By_{n}, Ax_{n}-Ax^{*}\bigr\rangle \\ &\quad = \Vert Ax_{n}-By_{n} \Vert ^{2}+ \bigl\Vert Ax_{n}-Ax^{*} \bigr\Vert ^{2}- \bigl\Vert By_{n}-Ax^{*} \bigr\Vert ^{2} \\ &\quad = \Vert Ax_{n}-By_{n} \Vert ^{2}+ \bigl\Vert Ax_{n}-Ax^{*} \bigr\Vert ^{2}- \bigl\Vert By_{n}-By^{*} \bigr\Vert ^{2} \end{aligned}$$

and

$$\begin{aligned} & 2\bigl\langle Ax_{n+1}-By_{n}, By_{n}-By^{*}\bigr\rangle \\ &\quad =- \Vert Ax_{n+1}-By_{n} \Vert ^{2}- \bigl\Vert By_{n}-By^{*} \bigr\Vert ^{2}+ \bigl\Vert Ax_{n+1}-By^{*} \bigr\Vert ^{2} \\ &\quad =- \Vert Ax_{n+1}-By_{n} \Vert ^{2}- \bigl\Vert By_{n}-By^{*} \bigr\Vert ^{2}+ \bigl\Vert Ax_{n+1}-Ax^{*} \bigr\Vert ^{2}. \end{aligned}$$

Altogether, we have

$$\begin{aligned} \bigl\Vert x_{n+1}-x^{*} \bigr\Vert ^{2} &\leq \bigl\Vert x_{n}-x^{*} \bigr\Vert ^{2}-\tau \bigl(2-\bigl(1+ \Vert A \Vert ^{2}\bigr)\tau \bigr) \Vert x_{n}-P_{C}x_{n} \Vert ^{2}-\tau \bigl\Vert Ax_{n}-Ax^{*} \bigr\Vert ^{2} \\ &\quad {} -\tau \bigl(1-\bigl(1+ \Vert A \Vert ^{2}\bigr)\tau \bigr) \Vert Ax_{n}-By_{n} \Vert ^{2}+\tau \bigl\Vert By_{n}-By^{*} \bigr\Vert ^{2} \\ &\leq \bigl\Vert x_{n}-x^{*} \bigr\Vert ^{2}-\tau \bigl(2-(1+c)\tau \bigr) \Vert x_{n}-P_{C}x_{n} \Vert ^{2}-\tau \bigl\Vert Ax_{n}-Ax^{*} \bigr\Vert ^{2} \\ &\quad {} -\tau \bigl(1-(1+c)\tau \bigr) \Vert Ax_{n}-By_{n} \Vert ^{2}+\tau \bigl\Vert By_{n}-By^{*} \bigr\Vert ^{2} \end{aligned}$$

and

$$\begin{aligned} \bigl\Vert y_{n+1}-y^{*} \bigr\Vert ^{2}&\leq \bigl\Vert y_{n}-y^{*} \bigr\Vert ^{2}-\tau \bigl(2-\bigl(1+ \Vert B \Vert ^{2}\bigr)\tau \bigr) \Vert y_{n}-P_{Q}y_{n} \Vert ^{2}+\tau \bigl\Vert Ax_{n+1}-Ax^{*} \bigr\Vert ^{2} \\ &\quad {} -\tau \bigl(1-\bigl(1+ \Vert B \Vert ^{2}\bigr)\tau \bigr) \Vert Ax_{n+1}-By_{n} \Vert ^{2}-\tau \bigl\Vert By_{n}-By^{*} \bigr\Vert ^{2} \\ &\leq \bigl\Vert y_{n}-y^{*} \bigr\Vert ^{2}-\tau \bigl(2-(1+c)\tau \bigr) \Vert y_{n}-P_{Q}y_{n} \Vert ^{2}+\tau \bigl\Vert Ax_{n+1}-Ax^{*} \bigr\Vert ^{2} \\ &\quad {} -\tau \bigl(1-(1+c)\tau \bigr) \Vert Ax_{n+1}-By_{n} \Vert ^{2}-\tau \bigl\Vert By_{n}-By^{*} \bigr\Vert ^{2}. \end{aligned}$$

Adding the two last inequalities, we obtain

3.3 $$\begin{aligned} & \bigl\Vert x_{n+1}-x^{*} \bigr\Vert ^{2}+ \bigl\Vert y_{n+1}-y^{*} \bigr\Vert ^{2} \\ &\quad \leq \bigl\Vert x_{n}-x^{*} \bigr\Vert ^{2}+ \bigl\Vert y_{n}-y^{*} \bigr\Vert ^{2}-\tau \bigl\Vert Ax_{n}-Ax^{*} \bigr\Vert ^{2}+\tau \bigl\Vert Ax_{n+1}-Ax^{*} \bigr\Vert ^{2} \\ &\qquad {} -\tau \bigl(2-(1+c)\tau \bigr) \bigl( \Vert x_{n}-P_{C}x_{n} \Vert ^{2}+ \Vert y_{n}-P_{Q}y_{n} \Vert ^{2}\bigr) \\ &\qquad {} -\tau \bigl(1-(1+c)\tau \bigr) \bigl( \Vert Ax_{n}-By_{n} \Vert ^{2}+ \Vert Ax_{n+1}-By_{n} \Vert ^{2}\bigr). \end{aligned}$$

Let $\varGamma_{n}(x^{*},y^{*})=\|x_{n}-x^{*}\|^{2}+\|y_{n}-y^{*}\|^{2}-\tau\|Ax_{n}-Ax^{*}\|^{2}$. Then $\tau\|Ax_{n}-Ax^{*}\|^{2}\leq\tau\|A\|^{2}\|x_{n}-x^{*}\|^{2}$, which implies

3.4 $$ \varGamma_{n}\bigl(x^{*},y^{*}\bigr)\geq\bigl(1-\tau \Vert A \Vert ^{2}\bigr) \bigl\Vert x_{n}-x^{*} \bigr\Vert ^{2}+ \bigl\Vert y_{n}-y^{*} \bigr\Vert ^{2} \geq0. $$

In view of (3.3), we obtain the following inequality:

3.5 $$\begin{aligned} \varGamma_{n+1}\bigl(x^{*},y^{*}\bigr)&\leq\varGamma_{n}\bigl(x^{*},y^{*} \bigr)-\tau \bigl(2-(1+c)\tau \bigr) \bigl( \Vert x_{n}-P_{C}x_{n} \Vert ^{2}+ \Vert y_{n}-P_{Q}y_{n} \Vert ^{2}\bigr) \\ &\quad {} -\tau \bigl(1-(1+c)\tau \bigr) \bigl( \Vert Ax_{n}-By_{n} \Vert ^{2}+ \Vert Ax_{n+1}-By_{n} \Vert ^{2}\bigr). \end{aligned}$$

This, together with (3.4), implies that the sequence $\{\varGamma_{n}(x^{*}, y^{*})\}$ is bounded and converges to some finite limit $\gamma(x^{*}, y^{*})$. By passing to the limit in (3.5) and by taking into account the assumption on *τ*, we finally obtain

3.6 $$ \lim_{n\rightarrow+\infty}\|x_{n}-P_{C}x_{n} \|=\lim_{n\rightarrow \infty}\|y_{n}-P_{Q}y_{n} \|=0 $$

and

3.7 $$ \lim_{n\rightarrow\infty}\|Ax_{n}-By_{n} \|=\lim_{n\rightarrow \infty}\|Ax_{n+1}-By_{n}\|=0. $$

We next prove that any weak cluster point of the sequence $\{(x_{n}, y_{n})\}$ is a solution of the SEP (1.4). Since $\{\varGamma_{n}(x^{*}, y^{*})\}$ is bounded, in view of (3.4), the sequences $\{x_{n}\}$ and $\{y_{n}\}$ are also bounded. Let *x̄* and *ȳ* be weak cluster points of the sequences $\{x_{n}\}$ and $\{y_{n}\}$, respectively. Without loss of generality, we assume that $x_{n}\rightharpoonup\bar{x}$ and $y_{n}\rightharpoonup\bar{y}$. Since $I-P_{C}$ and $I-P_{Q}$ are demiclosed at zero, from (3.6), we obtain $\bar{x}=P_{C}\bar{x}$ and $\bar{y}=P_{C}\bar{y}$, i.e., $\bar{x}\in C$ and $\bar{y}\in Q$. On the other hand, since $x_{n}\rightharpoonup\bar{x}$ and $y_{n}\rightharpoonup\bar{y}$, we deduce that $Ax_{n}-By_{n}\rightharpoonup A\bar{x}-B\bar{y}$. The weak lower semicontinuity of the squared norm implies

$$\|A\bar{x}-B\bar{y}\|^{2}\leq\liminf_{n\rightarrow \infty} \|Ax_{n}-By_{n}\|^{2}=0, $$

hence $(\bar{x}, \bar{y})\in S$.

We finally show the weak convergence of the sequence $\{(x_{n}, y_{n})\}$. Assume on the contrary that $(\hat{x},\hat{y})$ is another weak cluster point of $\{(x_{n},y_{n})\}$. By the definition of $\varGamma_{n}$, we have

$$\begin{aligned} \varGamma_{n}(\bar{x}, \bar{y})&=\varGamma_{n}(\hat{x}, \hat{y})+\| \bar{x}-\hat{x}\|^{2}+\|\bar{y}-\hat{y}\|^{2}-\tau\|A\bar{x}-A\hat{x}\|^{2} \\ &\quad {}+2\langle x_{n}-\hat{x}, \hat{x}-\bar{x}\rangle+2\langle y_{n}-\hat{y}, \hat{y}-\bar{y}\rangle-2\tau\langle Ax_{n}-A \hat{x}, A\hat{x}-A\bar{x}\rangle. \end{aligned}$$

By passing to the limit in the above, we obtain

$$\begin{aligned}& \gamma(\bar{x}, \bar{y})=\gamma(\hat{x}, \hat{y})+\|\bar{x}-\hat{x}\|^{2}+\| \bar{y}-\hat{y}\|^{2}-\tau\|A\bar{x}-A\hat{x}\|^{2}, \\& \gamma(\hat{x}, \hat{y})=\gamma(\bar{x}, \bar{y})+\|\bar{x}-\hat{x}\|^{2}+\| \bar{y}-\hat{y}\|^{2}-\tau\|A\bar{x}-A\hat{x}\|^{2}. \end{aligned}$$

By adding the last two equalities, we obtain

$$ \bigl(1-\tau\|A\|^{2}\bigr)\|\bar{x}-\hat{x}\|^{2}+\|\bar{y}- \hat{y}\|^{2}\leq0, $$

which clearly yields $\bar{x}=\hat{x}$ and $\bar{y}=\hat{y}$. This in particular implies that the weak cluster point of the sequence $\{(x_{n}, y_{n})\}$ is unique. Consequently, the whole sequence $\{(x_{n}, y_{n})\}$ converges weakly to a solution of problem (1.4). □

## A relaxed alternating CQ-algorithm

When *C* and *Q* are level sets, the projections in Algorithm 3.2 might be hard to be implemented (see [1, 8, 9, 13, 25, 26]). To overcome this difficulty, we propose a relaxed alternating CQ-algorithm, which is inspired by methods (1.8) and (3.2). In what follows, we will treat the SEP (1.4) under the following assumptions:

1. The sets *C* and *Q* are given by (1.6) and (1.7), respectively.
2. For any $x\in H_{1}$ and $y\in H_{2}$, at least one subgradient $\xi\in \partial c(x)$ and $\eta\in\partial q(y)$ can be calculated.

We now present a new relaxed alternative CQ algorithm for solving the SEP (1.4).

### Algorithm 4.1

Let $(x_{0}, y_{0})$ be arbitrary. Given $(x_{n}, y_{n})$, construct $(x_{n+1}, y_{n+1})$ via the formula

4.1 $$ \textstyle\begin{cases} x_{n+1}=x_{n}-\tau[(x_{n}-P_{C_{n}}x_{n})+A^{*}(Ax_{n}-By_{n})], \\ y_{n+1}=y_{n}-\tau[(y_{n}-P_{Q_{n}}y_{n})-B^{*}(Ax_{n+1}-By_{n})], \end{cases} $$

where $0<\tau<(1+c)^{-1}$ with $c=\max(\|A\|^{2}, \|B\|^{2})$, and $C_{n}$ and $Q_{n}$ are given as (1.9) and (1.10), respectively.

### Remark 4.2

By the definition of the subgradient, it is clear that $C\subseteq C_{n}$ and $Q\subseteq Q_{n}$ for all $n\geq0$. Since $C_{n}$ and $Q_{n}$ are both half-spaces, the projections onto $C_{n}$ and $Q_{n}$ can be easily calculated. Thus Algorithm 4.1 is easily implementable.

### Theorem 4.3

*Let* $\{(x_{n}, y_{n})\}$ *be the sequence generated by Algorithm* 4.1. *Then* $\{(x_{n}, y_{n})\}$ *converges weakly to a solution of the SEP* (1.4).

### Proof

Taking $(x^{*}, y^{*})\in S$, i.e., $x^{*}\in C$ (and thus $x^{*}\in C_{n}$), $y^{*}\in Q$ (and thus $y^{*}\in Q_{n}$), we have $Ax^{*}=By^{*}$. Let $\varGamma_{n} (x^{*},y^{*})=\|x_{n}-x^{*}\|^{2}+\|y_{n}-y^{*}\|^{2}-\tau\|Ax_{n}-Ax^{*}\|^{2}$. Similarly as in Theorem 3.3, we obtain the following inequality:

4.2 $$\begin{aligned} \varGamma_{n+1}\bigl(x^{*},y^{*}\bigr)&\leq\varGamma_{n}\bigl(x^{*},y^{*} \bigr)-\tau \bigl(2-(1+c)\tau \bigr) \bigl( \Vert x_{n}-P_{C_{n}}x_{n} \Vert ^{2}+ \Vert y_{n}-P_{Q_{n}}y_{n} \Vert ^{2}\bigr) \\ &\quad {} -\tau \bigl(1-(1+c)\tau \bigr) \bigl( \Vert Ax_{n}-By_{n} \Vert ^{2}+ \Vert Ax_{n+1}-By_{n} \Vert ^{2}\bigr). \end{aligned}$$

In addition, we have

4.3 $$ \varGamma_{n}\bigl(x^{*},y^{*}\bigr)\geq \bigl(1-\tau \Vert A \Vert ^{2}\bigr) \bigl\Vert x_{n}-x^{*} \bigr\Vert ^{2}+ \bigl\Vert y_{n}-y^{*} \bigr\Vert ^{2} \geq0. $$

It follows that the sequence $\{\varGamma_{n}(x^{*}, y^{*})\}$ is bounded and converges to some finite limit $\gamma(x^{*}, y^{*})$, which yields

4.4 $$ \lim_{n\rightarrow\infty}\|x_{n}-P_{C_{n}}x_{n} \|=\lim_{n\rightarrow \infty}\|y_{n}-P_{Q_{n}}y_{n} \|=0 $$

and

4.5 $$ \lim_{n\rightarrow\infty}\|Ax_{n}-By_{n}\|=\lim _{n\rightarrow \infty}\|Ax_{n+1}-By_{n}\|=0. $$

From (4.1), we obtain

$$\begin{aligned} \Vert x_{n+1}-x_{n} \Vert &= \bigl\Vert \tau \bigl[(x_{n}-P_{C_{n}}x_{n})+A^{*}(Ax_{n}-By_{n}) \bigr] \bigr\Vert \\ &\leq\tau\bigl( \Vert x_{n}-P_{C_{n}}x_{n} \Vert + \Vert A \Vert \Vert Ax_{n}-By_{n} \Vert \bigr)\rightarrow0 \end{aligned}$$

and

$$\begin{aligned} \Vert y_{n+1}-y_{n} \Vert &= \bigl\Vert \tau \bigl[(y_{n}-P_{Q_{n}}y_{n})-B^{*}(Ax_{n+1}-By_{n}) \bigr] \bigr\Vert \\ &\leq\tau\bigl( \Vert y_{n}-P_{Q_{n}}y_{n} \Vert + \Vert B \Vert \Vert Ax_{n+1}-By_{n} \Vert \bigr)\rightarrow0. \end{aligned}$$

We next prove that any weak cluster point of the sequence $\{(x_{n}, y_{n})\}$ is a solution of the SEP (1.4). Since $\{\varGamma_{n}(x^{*}, y^{*})\}$ is bounded, in view of (4.3), the sequences $\{x_{n}\}$ and $\{y_{n}\}$ are also bounded. Let *x̄* and *ȳ* be weak cluster points of the sequences $\{x_{n}\}$ and $\{y_{n}\}$, respectively. Without loss of generality, we assume that $x_{n}\rightharpoonup\bar{x}$ and $y_{n}\rightharpoonup\bar{y}$. Since *∂c* is bounded on bounded sets, there is a constant $\delta_{1} >0$ such that $\|\xi_{n}\|\leq\delta_{1}$ for all $n\geq0$. From (4.1), we have

$$ x_{n}-\frac{1}{\tau}(x_{n}-x_{n+1})+A^{*}(Ax_{n}-By_{n})=P_{C_{n}}x_{n} \in C_{n}. $$

This implies that

$$ c(x_{n})+\biggl\langle \xi_{n}, -\frac{1}{\tau}(x_{n}-x_{n+1})+A^{*}(Ax_{n}-By_{n}) \biggr\rangle \leq0. $$

Thus

$$\begin{aligned} c(x_{n})&\leq\biggl\langle \xi_{n}, \frac{1}{\tau}(x_{n}-x_{n+1})-A^{*}(Ax_{n}-By_{n}) \biggr\rangle \\ &\leq\frac{\delta_{1}}{\tau}\|x_{n}-x_{n+1}\|+ \delta_{1}\|A\|\|Ax_{n}-By_{n} \|\rightarrow0. \end{aligned}$$

The weak lower semicontinuity of *c* leads to

$$ c(\bar{x})\leq\liminf_{n\rightarrow\infty}c(x_{n})\leq0, $$

and therefore $\bar{x}\in C$. Likewise, since *∂q* is bounded on bounded sets, there is a constant $\delta_{2} >0$ such that $\|\eta_{n}\|\leq\delta_{2}$ for all $n\geq0$. From (4.1), we have

$$ y_{n}-\frac{1}{\tau}(y_{n}-y_{n+1})-B^{*}(Ax_{n+1}-By_{n})=P_{Q_{n}}y_{n} \in Q_{n}. $$

This implies that

$$ q(y_{n})+\biggl\langle \eta_{n}, -\frac{1}{\tau}(y_{n}-y_{n+1})-B^{*}(Ax_{n+1}-By_{n}) \biggr\rangle \leq0. $$

Hence

$$\begin{aligned} q(y_{n})&\leq\biggl\langle \eta_{n}, \frac{1}{\tau}(y_{n}-y_{n+1})+B^{*}(Ax_{n+1}-By_{n}) \biggr\rangle \\ &\leq \frac{\delta_{2}}{\tau}\|y_{n}-y_{n+1}\|+ \delta_{2}\|B\|\|Ax_{n+1}-By_{n} \|\rightarrow0. \end{aligned}$$

Again, the weak lower semicontinuity of *q* leads to

$$ q(\bar{y})\leq\liminf_{n\rightarrow\infty}q(y_{n})\leq0, $$

and therefore $\bar{y}\in Q$. Furthermore, the weak convergence of $\{Ax_{n}-By_{n}\}$ to $A\bar{x}-B\bar{y}$ and the weak lower semicontinuity of the squared norm imply

$$ \|A\bar{x}-B\bar{y}\|^{2}\leq\liminf_{n\rightarrow \infty} \|Ax_{n}-By_{n}\|^{2}=0. $$

Hence $(\bar{x}, \bar{y})\in S$.

The proof of the uniqueness of the weak cluster point is analogous to that of Theorem 3.3. Therefore, the whole sequence $\{(x_{n}, y_{n})\}$ converges weakly to a solution of problem (1.4). This completes the proof. □

## A strongly convergent algorithm

As we see from the previous section, the sequence generated by Algorithm 4.1 is only weakly convergent. So, the aim of this section is to modify Algorithm 4.1 so that it generates a strongly convergent sequence. This provides an affirmative answer to the open question raised by Moudafi [10].

### Algorithm 5.1

Let $(u, v)\in H_{1}\times H_{2}$ be fixed and start with an initial guess $(x_{0}, y_{0})\in H_{1}\times H_{2}$. Given $(x_{n}, y_{n})$, construct $(x_{n+1}, y_{n+1})$ via the formula

5.1 $$ \textstyle\begin{cases} u_{n}=x_{n}-\tau[(x_{n}-P_{C_{n}}x_{n})+A^{*}(Ax_{n}-By_{n})], \\ x_{n+1}=\alpha_{n} u+(1-\alpha_{n})u_{n}, \\ v_{n}=y_{n}-\tau[(y_{n}-P_{Q_{n}}y_{n})-B^{*}(Ax_{n+1}-By_{n})], \\ y_{n+1}=\alpha_{n} v+(1-\alpha_{n})v_{n}, \end{cases} $$

where $\{\alpha_{n}\}$ is a sequence in $[0,1]$, $0<\tau<(1+c)^{-1}$ with $c=\max(\|A\|^{2}, \|B\|^{2})$, and $C_{n}$ and $Q_{n}$ are given as (1.9) and (1.10), respectively.

### Theorem 5.2

*Let* $\{(x_{n}, y_{n})\}$ *be the sequence generated by Algorithm* 5.1. *If* $\{\alpha_{n}\}$ *satisfies the following conditions*:

$$\begin{aligned} \lim_{n\rightarrow\infty}\alpha_{n}=0 \quad \textit{and}\quad \sum _{n=1}^{\infty}\alpha_{n}=\infty, \end{aligned}$$

*then* $\{(x_{n}, y_{n})\}$ *converges strongly to a solution* $(x^{*}, y^{*})$ *of the SEP* (1.4), *where* $(x^{*}, y^{*})=P_{S}(u,v)$.

### Proof

Since $(x^{*}, y^{*})=P_{S}(u,v)\in S$, we have $x^{*}\in C$ (and thus $x^{*}\in C_{n}$), $y^{*}\in Q$ (and thus $y^{*}\in Q_{n}$), $Ax^{*}=By^{*}$. In what follows, we divide the proof into four steps.

*Step* 1. We prove that the sequences $\{x_{n}\}$ and $\{y_{n}\}$ are bounded. By the same argument as in the proof of Theorem 3.3, we arrive at

5.2 $$\begin{aligned} & \bigl\Vert u_{n}-x^{*} \bigr\Vert ^{2}+ \bigl\Vert v_{n}-y^{*} \bigr\Vert ^{2} \\ &\quad \leq \bigl\Vert x_{n}-x^{*} \bigr\Vert ^{2}+ \bigl\Vert y_{n}-y^{*} \bigr\Vert ^{2}-\tau \bigl\Vert Ax_{n}-Ax^{*} \bigr\Vert ^{2}+\tau \bigl\Vert Ax_{n+1}-Ax^{*} \bigr\Vert ^{2} \\ &\qquad {} -\tau \bigl(2-(1+c)\tau \bigr) \bigl( \Vert x_{n}-P_{C_{n}}x_{n} \Vert ^{2}+ \Vert y_{n}-P_{Q_{n}}y_{n} \Vert ^{2}\bigr) \\ & \qquad {}-\tau \bigl(1-(1+c)\tau \bigr) \bigl( \Vert Ax_{n}-By_{n} \Vert ^{2}+ \Vert Ax_{n+1}-By_{n} \Vert ^{2}\bigr). \end{aligned}$$

In view of (5.1) and the convexity of the squared norm, we obtain

$$\begin{aligned} & \bigl\Vert x_{n+1}-x^{*} \bigr\Vert ^{2}+ \bigl\Vert y_{n+1}-y^{*} \bigr\Vert ^{2} \\ &\quad = \bigl\Vert \alpha_{n} \bigl(u-x^{*}\bigr)+(1- \alpha_{n}) \bigl(u_{n}-x^{*}\bigr) \bigr\Vert ^{2}+ \bigl\Vert \alpha_{n} \bigl(v-y^{*}\bigr)+(1- \alpha_{n}) \bigl(v_{n}-y^{*}\bigr) \bigr\Vert ^{2} \\ &\quad \leq \alpha_{n} \bigl\Vert u-x^{*} \bigr\Vert ^{2}+(1-\alpha_{n}) \bigl\Vert u_{n}-x^{*} \bigr\Vert ^{2}+\alpha_{n} \bigl\Vert v-y^{*} \bigr\Vert ^{2}+(1-\alpha_{n}) \bigl\Vert v_{n}-y^{*} \bigr\Vert ^{2} \\ &\quad =\alpha_{n}\bigl( \bigl\Vert u-x^{*} \bigr\Vert ^{2}+ \bigl\Vert v-y^{*} \bigr\Vert ^{2}\bigr)+(1- \alpha_{n}) \bigl( \bigl\Vert u_{n}-x^{*} \bigr\Vert ^{2}+ \bigl\Vert v_{n}-y^{*} \bigr\Vert ^{2} \bigr). \end{aligned}$$

This, along with (5.2), implies that

5.3 $$\begin{aligned} & \bigl\Vert x_{n+1}-x^{*} \bigr\Vert ^{2}+ \bigl\Vert y_{n+1}-y^{*} \bigr\Vert ^{2} \\ &\quad \leq\alpha_{n}\bigl( \bigl\Vert u-x^{*} \bigr\Vert ^{2}+ \bigl\Vert v-y^{*} \bigr\Vert ^{2}\bigr)+(1- \alpha_{n}) \bigl[ \bigl\Vert x_{n}-x^{*} \bigr\Vert ^{2}+ \bigl\Vert y_{n}-y^{*} \bigr\Vert ^{2} \\ &\qquad {} -\tau \bigl\Vert Ax_{n}-Ax^{*} \bigr\Vert ^{2}+\tau \bigl\Vert Ax_{n+1}-Ax^{*} \bigr\Vert ^{2} \\ & \qquad {}-\tau \bigl(2-(1+c)\tau \bigr) \bigl( \Vert x_{n}-P_{C_{n}}x_{n} \Vert ^{2}+ \Vert y_{n}-P_{Q_{n}}y_{n} \Vert ^{2}\bigr) \\ &\qquad {} -\tau \bigl(1-(1+c)\tau \bigr) \bigl( \Vert Ax_{n}-By_{n} \Vert ^{2}+ \Vert Ax_{n+1}-By_{n} \Vert ^{2}\bigr) \bigr] \\ &\quad \leq\alpha_{n}\bigl( \bigl\Vert u-x^{*} \bigr\Vert ^{2}+ \bigl\Vert v-y^{*} \bigr\Vert ^{2}\bigr)+\tau \bigl\Vert Ax_{n+1}-Ax^{*} \bigr\Vert ^{2} \\ &\qquad {} +(1-\alpha_{n}) \bigl[ \bigl\Vert x_{n}-x^{*} \bigr\Vert ^{2}+ \bigl\Vert y_{n}-y^{*} \bigr\Vert ^{2}-\tau \bigl\Vert Ax_{n}-Ax^{*} \bigr\Vert ^{2} \\ &\qquad {} -\tau \bigl(2-(1+c)\tau \bigr) \bigl( \Vert x_{n}-P_{C_{n}}x_{n} \Vert ^{2}+ \Vert y_{n}-P_{Q_{n}}y_{n} \Vert ^{2}\bigr) \\ & \qquad {}-\tau \bigl(1-(1+c)\tau \bigr) \bigl( \Vert Ax_{n}-By_{n} \Vert ^{2}+ \Vert Ax_{n+1}-By_{n} \Vert ^{2}\bigr) \bigr]. \end{aligned}$$

Now, by setting

$$\varGamma_{n} \bigl(x^{*},y^{*}\bigr)= \bigl\Vert x_{n}-x^{*} \bigr\Vert ^{2}+ \bigl\Vert y_{n}-y^{*} \bigr\Vert ^{2}-\tau \bigl\Vert Ax_{n}-Ax^{*} \bigr\Vert ^{2}, $$

we have

5.4 $$ \varGamma_{n}\bigl(x^{*},y^{*}\bigr)\geq \bigl(1-\tau \Vert A \Vert ^{2}\bigr) \bigl\Vert x_{n}-x^{*} \bigr\Vert ^{2}+ \bigl\Vert y_{n}-y^{*} \bigr\Vert ^{2} \geq0. $$

In view of (5.3), we conclude that

$$\begin{aligned} \varGamma_{n+1}\bigl(x^{*},y^{*}\bigr)&\leq (1-\alpha_{n}) \varGamma_{n}\bigl(x^{*},y^{*}\bigr)+\alpha_{n}\bigl( \bigl\Vert u-x^{*} \bigr\Vert ^{2}+ \bigl\Vert v-y^{*} \bigr\Vert ^{2} \bigr) \\ &\quad {} -(1-\alpha_{n}) \bigl[\tau \bigl(2-(1+c)\tau \bigr) \bigl( \Vert x_{n}-P_{C_{n}}x_{n} \Vert ^{2}+ \Vert y_{n}-P_{Q_{n}}y_{n} \Vert ^{2} \bigr) \\ & \quad {}+\tau \bigl(1-(1+c)\tau \bigr) \bigl( \Vert Ax_{n}-By_{n} \Vert ^{2}+ \Vert Ax_{n+1}-By_{n} \Vert ^{2}\bigr) \bigr]. \end{aligned}$$

This implies

$$\begin{aligned} \varGamma_{n+1}\bigl(x^{*},y^{*}\bigr)&\leq (1-\alpha_{n}) \varGamma_{n}\bigl(x^{*},y^{*}\bigr)+\alpha_{n}\bigl( \bigl\Vert u-x^{*} \bigr\Vert ^{2}+ \bigl\Vert v-y^{*} \bigr\Vert ^{2} \bigr) \\ &\leq\max\bigl\{ \varGamma_{n}\bigl(x^{*},y^{*}\bigr), \bigl\Vert u-x^{*} \bigr\Vert ^{2}+ \bigl\Vert v-y^{*} \bigr\Vert ^{2}\bigr\} . \end{aligned}$$

By induction, we obtain

$$ \varGamma_{n+1}\bigl(x^{*},y^{*}\bigr)\leq\max\bigl\{ \varGamma_{0} \bigl(x^{*},y^{*}\bigr), \bigl\Vert u-x^{*} \bigr\Vert ^{2}+ \bigl\Vert v-y^{*} \bigr\Vert ^{2}\bigr\} $$

for all $n\geq0$. This implies that the sequence $\{\varGamma_{n}(x^{*}, y^{*})\}$ is bounded. Hence, in view of (5.4), the sequences $\{x_{n}\}$ and $\{y_{n}\}$ are bounded, too.

*Step* 2. We show that the following inequality holds:

5.5 $$ \varGamma_{n+1}\bigl(x^{*}, y^{*}\bigr)\leq(1- \alpha_{n})\varGamma_{n}\bigl(x^{*}, y^{*}\bigr)+ \alpha_{n}\delta_{n}, $$

where

$$\begin{aligned} \delta_{n}&=2\bigl(\bigl\langle u-x^{*}, x_{n+1}-x^{*}\bigr\rangle +\bigl\langle v-y^{*}, y_{n+1}-y^{*}\bigr\rangle \bigr) \\ &\quad {} -\frac{(1-\alpha_{n})}{\alpha_{n}} \bigl[\tau \bigl(2-(1+c)\tau \bigr) \bigl( \Vert x_{n}-P_{C_{n}}x_{n} \Vert ^{2}+ \Vert y_{n}-P_{Q_{n}}y_{n} \Vert ^{2}\bigr) \\ &\quad {} +\tau \bigl(1-(1+c)\tau \bigr) \bigl( \Vert Ax_{n}-By_{n} \Vert ^{2}+ \Vert Ax_{n+1}-By_{n} \Vert ^{2}\bigr) \bigr]. \end{aligned}$$

Indeed, by Lemma 2.5, we have

$$\begin{aligned} & \bigl\Vert x_{n+1}-x^{*} \bigr\Vert ^{2}+ \bigl\Vert y_{n+1}-y^{*} \bigr\Vert ^{2} \\ &\quad = \bigl\Vert \alpha_{n} \bigl(u-x^{*}\bigr)+(1- \alpha_{n}) \bigl(u_{n}-x^{*}\bigr) \bigr\Vert ^{2}+ \bigl\Vert \alpha_{n} \bigl(v-y^{*}\bigr)+(1- \alpha_{n}) \bigl(v_{n}-y^{*}\bigr) \bigr\Vert ^{2} \\ &\quad \leq (1-\alpha_{n}) \bigl\Vert u_{n}-x^{*} \bigr\Vert ^{2}+2\alpha_{n}\bigl\langle u-x^{*}, x_{n+1}-x^{*}\bigr\rangle \\ &\qquad {} +(1-\alpha_{n}) \bigl\Vert v_{n}-y^{*} \bigr\Vert ^{2}+2\alpha_{n}\bigl\langle v-y^{*}, y_{n+1}-y^{*}\bigr\rangle \\ &\quad = (1-\alpha_{n}) \bigl( \bigl\Vert u_{n}-x^{*} \bigr\Vert ^{2}+ \bigl\Vert v_{n}-y^{*} \bigr\Vert ^{2}\bigr) \\ &\qquad {} +2\alpha_{n}\bigl(\bigl\langle u-x^{*}, x_{n+1}-x^{*} \bigr\rangle +\bigl\langle v-y^{*}, y_{n+1}-y^{*}\bigr\rangle \bigr). \end{aligned}$$

Again from (5.2), we obtain

$$\begin{aligned} & \bigl\Vert x_{n+1}-x^{*} \bigr\Vert ^{2}+ \bigl\Vert y_{n+1}-y^{*} \bigr\Vert ^{2} \\ &\quad \leq (1-\alpha_{n}) \bigl[ \bigl\Vert x_{n}-x^{*} \bigr\Vert ^{2}+ \bigl\Vert y_{n}-y^{*} \bigr\Vert ^{2}-\tau \bigl\Vert Ax_{n}-Ax^{*} \bigr\Vert ^{2}+\tau \bigl\Vert Ax_{n+1}-By^{*} \bigr\Vert ^{2} \\ &\qquad {} -\tau \bigl(2-(1+c)\tau \bigr) \bigl( \Vert x_{n}-P_{C_{n}}x_{n} \Vert ^{2}+ \Vert y_{n}-P_{Q_{n}}y_{n} \Vert ^{2}\bigr) \\ & \qquad {}-\tau \bigl(1-(1+c)\tau \bigr) \bigl( \Vert Ax_{n}-By_{n} \Vert ^{2}+ \Vert Ax_{n+1}-By_{n} \Vert ^{2}\bigr) \bigr] \\ &\qquad {} +2\alpha_{n}\bigl(\bigl\langle u-x^{*}, x_{n+1}-x^{*} \bigr\rangle +\bigl\langle v-y^{*}, y_{n+1}-y^{*}\bigr\rangle \bigr) \\ &\quad \leq (1-\alpha_{n}) \bigl( \bigl\Vert x_{n}-x^{*} \bigr\Vert ^{2}+ \bigl\Vert y_{n}-y^{*} \bigr\Vert ^{2}-\tau \bigl\Vert Ax_{n}-Ax^{*} \bigr\Vert ^{2}\bigr)+\tau \bigl\Vert Ax_{n+1}-By^{*} \bigr\Vert ^{2} \\ &\qquad {} -(1-\alpha_{n}) \bigl[\tau \bigl(2-(1+c)\tau \bigr) \bigl( \Vert x_{n}-P_{C_{n}}x_{n} \Vert ^{2}+ \Vert y_{n}-P_{Q_{n}}y_{n} \Vert ^{2} \bigr) \\ &\qquad {} +\tau \bigl(1-(1+c)\tau \bigr) \bigl( \Vert Ax_{n}-By_{n} \Vert ^{2}+ \Vert Ax_{n+1}-By_{n} \Vert ^{2}\bigr) \bigr] \\ &\qquad {} +2\alpha_{n}\bigl(\bigl\langle u-x^{*}, x_{n+1}-x^{*} \bigr\rangle +\bigl\langle v-y^{*}, y_{n+1}-y^{*}\bigr\rangle \bigr). \end{aligned}$$

This implies

$$\begin{aligned} \varGamma_{n+1}\bigl(x^{*}, y^{*}\bigr) &\leq (1-\alpha_{n}) \varGamma_{n}\bigl(x^{*}, y^{*}\bigr) \\ &\quad {} -(1-\alpha_{n}) \bigl[\tau \bigl(2-(1+c)\tau \bigr) \bigl( \Vert x_{n}-P_{C_{n}}x_{n} \Vert ^{2}+ \Vert y_{n}-P_{Q_{n}}y_{n} \Vert ^{2} \bigr) \\ &\quad {} +\tau \bigl(1-(1+c)\tau \bigr) \bigl( \Vert Ax_{n}-By_{n} \Vert ^{2}+ \Vert Ax_{n+1}-By_{n} \Vert ^{2}\bigr) \bigr] \\ &\quad {} +2\alpha_{n}\bigl(\bigl\langle u-x^{*}, x_{n+1}-x^{*} \bigr\rangle +\bigl\langle v-y^{*}, y_{n+1}-y^{*}\bigr\rangle \bigr) \\ &=(1-\alpha_{n})\varGamma_{n}\bigl(x^{*}, y^{*}\bigr) + \alpha_{n} \biggl\{ 2\bigl(\bigl\langle u-x^{*}, x_{n+1}-x^{*}\bigr\rangle +\bigl\langle v-y^{*}, y_{n+1}-y^{*}\bigr\rangle \bigr) \\ &\quad {} -\frac{(1-\alpha_{n})}{\alpha_{n}} \bigl[\tau \bigl(2-(1+c)\tau \bigr) \bigl( \Vert x_{n}-P_{C_{n}}x_{n} \Vert ^{2}+ \Vert y_{n}-P_{Q_{n}}y_{n} \Vert ^{2}\bigr) \\ &\quad {} +\tau \bigl(1-(1+c)\tau \bigr) \bigl( \Vert Ax_{n}-By_{n} \Vert ^{2}+ \Vert Ax_{n+1}-By_{n} \Vert ^{2}\bigr) \bigr] \biggr\} . \end{aligned}$$

Hence, the desired inequality follows at once.

*Step* 3. We show that $\limsup_{n\rightarrow \infty}\delta_{n}$ is finite. Since $\{x_{n}\}$ and $\{y_{n}\}$ are bounded, we have

$$\begin{aligned} \delta_{n}&\leq2\bigl(\bigl\langle u-x^{*}, x_{n+1}-x^{*}\bigr\rangle +\bigl\langle v-y^{*}, y_{n+1}-y^{*}\bigr\rangle \bigr) \\ &\leq2\bigl( \bigl\Vert u-x^{*} \bigr\Vert \bigl\Vert x_{n+1}-x^{*} \bigr\Vert + \bigl\Vert v-y^{*} \bigr\Vert \bigl\Vert y_{n+1}-y^{*} \bigr\Vert \bigr)< \infty. \end{aligned}$$

This implies that $\limsup_{n\rightarrow \infty}\delta_{n}<\infty$. We now show $\limsup_{n\rightarrow \infty}\delta_{n}\geq-1$ by contradiction. If we assume on the contrary that $\limsup_{n\rightarrow\infty}\delta_{n}<-1$, then there exists $n_{0}$ such that $\delta_{n}\leq-1$ for all $n\geq n_{0}$. It then follows from (5.5) that

$$\begin{aligned} \varGamma_{n+1}\bigl(x^{*}, y^{*}\bigr)&\leq(1-\alpha_{n}) \varGamma_{n}\bigl(x^{*}, y^{*}\bigr)+\alpha_{n} \delta_{n} \\ &\leq(1-\alpha_{n})\varGamma_{n}\bigl(x^{*}, y^{*}\bigr)- \alpha_{n} \\ &=\varGamma_{n}\bigl(x^{*}, y^{*}\bigr)-\alpha_{n}\bigl( \varGamma_{n}\bigl(x^{*}, y^{*}\bigr)+1\bigr) \\ &\leq\varGamma_{n}\bigl(x^{*}, y^{*}\bigr)-\alpha_{n} \end{aligned}$$

for all $n\geq n_{0}$. By induction, we have

$$ \varGamma_{n+1}\bigl(x^{*}, y^{*}\bigr)\leq\varGamma_{n_{0}}\bigl(x^{*}, y^{*} \bigr)-\sum_{i=n_{0}}^{n}\alpha_{i}. $$

Since $\sum_{i=n_{0}}^{\infty}\alpha_{i}=\infty$, there exists $N>n_{0}$ such that $\sum_{i=n_{0}}^{N}\alpha_{i}>\varGamma_{n_{0}}(x^{*}, y^{*})$. Therefore, we have

$$\varGamma_{N+1}\bigl(x^{*}, y^{*}\bigr)\leq\varGamma_{n_{0}}\bigl(x^{*}, y^{*} \bigr)-\sum_{i=n_{0}}^{N}\alpha_{i}< 0, $$

which clearly contradicts the fact that $\varGamma_{n}(x^{*}, y^{*})$ is a nonnegative real sequence. Thus, $\limsup_{n\rightarrow\infty}\delta_{n}\geq-1$ and it is finite.

*Step* 4. We show that $\limsup_{n\rightarrow \infty}\delta_{n}\leq0$ and $\{(x_{n}, y_{n})\}$ converges strongly to $(x^{*}, y^{*})$. Since $\limsup_{n\rightarrow\infty}\delta_{n}$ is finite, we can take a subsequence $\{n_{k}\}$ such that

5.6 $$\begin{aligned} \limsup_{n\rightarrow \infty}\delta_{n}&=\lim_{k\rightarrow \infty} \delta_{n_{k}} \\ &=\lim_{k\rightarrow\infty} \biggl\{ 2\bigl(\bigl\langle u-x^{*}, x_{n_{k}+1}-x^{*}\bigr\rangle +\bigl\langle v-y^{*}, y_{n_{k}+1}-y^{*}\bigr\rangle \bigr) \\ &\quad {} -\frac{(1-\alpha_{n_{k}})}{\alpha_{n_{k}}} \bigl[\tau \bigl(2-(1+c)\tau \bigr) \bigl( \|x_{n_{k}}-P_{C_{n_{k}}}x_{n_{k}}\|^{2}+ \|y_{n_{k}}-P_{Q_{n_{k}}}y_{n_{k}}\|^{2}\bigr) \\ &\quad {} +\tau \bigl(1-(1+c)\tau \bigr) \bigl(\|Ax_{n_{k}}-By_{n_{k}} \|^{2}+\| Ax_{n_{k}+1}-By_{n_{k}}\|^{2}\bigr) \bigr] \biggr\} . \end{aligned}$$

Since $\langle u-x^{*}, x_{n+1}-x^{*}\rangle$ and $\langle v-y^{*}, y_{n+1}-y^{*}\rangle$ are bounded, without loss of generality, we may assume the existence of the limits

$$ \lim_{k\rightarrow\infty}\bigl\langle u-x^{*}, x_{n_{k}+1}-x^{*}\bigr\rangle \quad \text{and}\quad \lim_{k\rightarrow\infty}\bigl\langle v-y^{*}, y_{n_{k}+1}-y^{*}\bigr\rangle . $$

Hence, from (5.6), the following limit also exists:

$$\begin{aligned} &\lim_{k\rightarrow\infty} \frac{(1-\alpha_{n_{k}})}{\alpha_{n_{k}}} \bigl[\tau \bigl(2-(1+c)\tau \bigr) \bigl(\| x_{n_{k}}-P_{C_{n_{k}}}x_{n_{k}}\|^{2}+ \|y_{n_{k}}-P_{Q_{n_{k}}}y_{n_{k}}\|^{2}\bigr) \\ &\quad {}+\tau \bigl(1-(1+c)\tau \bigr) \bigl(\|Ax_{n_{k}}-By_{n_{k}} \|^{2}+\| Ax_{n_{k}+1}-By_{n_{k}}\|^{2}\bigr) \bigr]. \end{aligned}$$

Since $\lim_{k\rightarrow\infty}\alpha_{n_{k}}=0$, we get $\lim_{k\rightarrow \infty}\frac{1-\alpha_{n_{k}}}{\alpha_{n_{k}}}=\infty$. This implies that

$$\begin{aligned} &\lim_{k\rightarrow\infty} \bigl[\tau \bigl(2-(1+c)\tau \bigr) \bigl(\| x_{n_{k}}-P_{C_{n_{k}}}x_{n_{k}}\|^{2}+ \|y_{n_{k}}-P_{Q_{n_{k}}}y_{n_{k}}\|^{2}\bigr) \\ &\quad {}+\tau \bigl(1-(1+c)\tau \bigr) \bigl(\|Ax_{n_{k}}-By_{n_{k}} \|^{2}+\| Ax_{n_{k}+1}-By_{n_{k}}\|^{2}\bigr) \bigr]=0. \end{aligned}$$

So, by taking into account the assumption on *τ*, we have

$$ \lim_{k\rightarrow \infty}\|x_{n_{k}}-P_{C_{n_{k}}}x_{n_{k}} \|=\lim_{k\rightarrow \infty}\|y_{n_{k}}-P_{Q_{n_{k}}}y_{n_{k}} \|=0 $$

and

$$ \lim_{k\rightarrow\infty}\|Ax_{n_{k}}-By_{n_{k}}\|=\lim _{k\rightarrow \infty}\|Ax_{n_{k}+1}-By_{n_{k}}\|=0. $$

From (5.1), we deduce that

$$\begin{aligned} \lim_{k\rightarrow \infty} \Vert u_{n_{k}}-x_{n_{k}} \Vert &=\lim_{k\rightarrow\infty}\tau \bigl\Vert (x_{n_{k}}-P_{C_{n_{k}}}x_{n_{k}})+A^{*}(Ax_{n_{k}}-By_{n_{k}}) \bigr\Vert \\ &\leq\tau \lim_{k\rightarrow\infty}\bigl( \Vert x_{n_{k}}-P_{C_{n_{k}}}x_{n_{k}} \Vert + \Vert A \Vert \Vert Ax_{n_{k}}-By_{n_{k}} \Vert \bigr)=0 \end{aligned}$$

and

$$\begin{aligned} \lim_{k\rightarrow \infty} \Vert v_{n_{k}}-y_{n_{k}} \Vert &=\lim_{k\rightarrow\infty}\tau \bigl\Vert (y_{n_{k}}-P_{Q_{n_{k}}}y_{n_{k}})-B^{*}(Ax_{n_{k}+1}-By_{n_{k}}) \bigr\Vert \\ &\leq\tau \lim_{k\rightarrow\infty}\bigl( \Vert y_{n_{k}}-P_{Q_{n_{k}}}y_{n_{k}} \Vert + \Vert B \Vert \Vert Ax_{n_{k}+1}-By_{n_{k}} \Vert \bigr)=0. \end{aligned}$$

Similarly as in the proof of Theorem 4.3, we conclude that any weak cluster point of $\{(x_{n_{k}}, y_{n_{k}})\}$ belongs to *S*.

Since the sequences $\{x_{n}\}$ and $\{y_{n}\}$ are bounded, one gets

$$\begin{aligned} \lim_{k\rightarrow \infty} \Vert x_{n_{k}+1}-x_{n_{k}} \Vert &=\lim_{k\rightarrow\infty} \bigl\Vert \alpha _{n_{k}}(u-x_{n_{k}})+(1- \alpha_{n_{k}}) (u_{n_{k}}-x_{n_{k}}) \bigr\Vert \\ &\leq \lim_{k\rightarrow\infty}\bigl(\alpha_{n_{k}} \Vert u-x_{n_{k}} \Vert + \Vert u_{n_{k}}-x_{n_{k}} \Vert \bigr)=0 \end{aligned}$$

and

$$\begin{aligned} \lim_{k\rightarrow\infty} \Vert y_{n_{k}+1}-y_{n_{k}} \Vert &=\lim_{k\rightarrow\infty } \bigl\Vert \alpha_{n_{k}}(v-y_{n_{k}})+(1- \alpha_{n_{k}}) (v_{n_{k}}-y_{n_{k}}) \bigr\Vert \\ &\leq \lim_{k\rightarrow\infty}\bigl(\alpha_{n_{k}} \Vert v-y_{n_{k}} \Vert + \Vert v_{n_{k}}-y_{n_{k}} \Vert \bigr)=0. \end{aligned}$$

This implies that any weak cluster point of $\{(x_{n_{k}+1}, y_{n_{k}+1})\}$ also belongs to *S*. Without loss of generality, we assume that $\{(x_{n_{k}+1}, y_{n_{k}+1})\}$ converges weakly to $(\hat{x}, \hat{y})\in S$. Now by (5.6), Lemma 2.2 and the fact that $(x^{*}, y^{*})=P_{S}(u,v)$, we obtain

$$\begin{aligned} \limsup_{n\rightarrow\infty}\delta_{n} &\leq \lim _{k\rightarrow\infty}2\bigl(\bigl\langle u-x^{*}, x_{n_{k}+1}-x^{*}\bigr\rangle +\bigl\langle v-y^{*}, y_{n_{k}+1}-y^{*}\bigr\rangle \bigr) \\ &=2\bigl(\bigl\langle u-x^{*}, \hat{x}-x^{*}\bigr\rangle +\bigl\langle v-y^{*}, \hat{y}-y^{*}\bigr\rangle \bigr)\leq0. \end{aligned}$$

Applying Lemma 2.6 to (5.5), we have $\lim_{n\rightarrow\infty}\varGamma_{n}(x^{*}, y^{*})=0$. Finally, by (5.4), we infer that

$$ \lim_{n\rightarrow\infty} \bigl\Vert x_{n}-x^{*} \bigr\Vert =0 \quad \text{and}\quad \lim_{n\rightarrow\infty} \bigl\Vert y_{n}-y^{*} \bigr\Vert =0, $$

which ends the proof. □

## Numerical results

In this section, we verify the feasibility and efficiency of our algorithms through an example. The whole codes are written in Matlab R2012b on a personal computer with Inter(R) Core(TM) i5-4590 CPU, 3.30 GHz and 4 GB RAM.

### Example 6.1

Let $H_{1}=H_{2}=H_{3}=\mathbb{R}^{3}$,

$$A=\left[\begin{array}{ccc} \sqrt{5} & 0 & 0\\ 0 & 5 & 0\\ 0 & 0 & 1 \end{array}\right],\;B=\left[\begin{array}{ccc} 1 & 0 & 0\\ 0 & 1 & 0\\ 0 & 0 & 1 \end{array}\right],$$

$C=\{x\in\mathbb{R}^{3}\mid x=(u, v, w)^{T}, v^{2}+w^{2}-1\leq0\}$, and $Q=\{ y\in\mathbb{R}^{3}\mid y=(u, v, w)^{T}, u^{2}-v+5\leq0\}$. Find $x\in C$, $y\in Q$ such that $Ax=By$.

It is easy to verify that this problem has a unique solution $(\bar{x}, \bar{y})\in\mathbb{R}^{3}\times \mathbb{R}^{3}$, where $\bar{x}=(0, 1, 0)^{T}$, $\bar{y}=(0, 5, 0)^{T}$. In the experiments, we take $\gamma=0.9\times\min(\frac{1}{\|A\|^{2}},\frac{1}{\|B\|^{2}})$ in RACQA algorithm (1.8) and $\tau=0.9\times(1+c)^{-1}$ with $c=\max(\|A\|^{2}, \|B\|^{2})$ in Algorithm 4.1. The stopping criterion is $\|x_{k+1}-x_{k}\|+\|y_{k+1}-y_{k}\|<10^{-3}$ and $\|Ax_{k}-By_{k}\|<10^{-3}$. The numerical results can be seen from Tables 1–3. It is worth noting that the initial point in Table 3 is generated randomly. From Tables 1–3, we can see that the CPU time and iteration number of Algorithm 4.1 are less than that of RACQA algorithm (1.8).

**Table 1:** Numerical results of Example 6.1

Initial point: $x_{0}=(1,1,1)^{T}$, $y_{0}=(0,0,0)^{T}$			
Algorithm	Time (s)	No. Iterations	Approximate solution $(x^{*},y^{*})$
RACQA (1.8)	0.34021	7357	$x^{*}=(0.0130,1.000,0.0079)^{T}$
			$y^{*}=(0.0291,5.0008,0.0079)^{T}$
Algorithm 4.1	0.26312	5847	$x^{*}=(0.0001,0.9996,0.1028)^{T}$
			$y^{*}=(0.0002,4.9990,0.1030)^{T}$

**Table 2:** Numerical results of Example 6.1

Initial point: $x_{0}=(5,5,5)^{T}$, $y_{0}=(1,1,1)^{T}$			
Algorithm	Time (s)	No. Iterations	Approximate solution $(x^{*},y^{*})$
RACQA (1.8)	0.32586	7010	$x^{*}=(0.0120,0.9999,0.0106)^{T}$
			$y^{*}=(0.0268,5.0007,0.0106)^{T}$
Algorithm 4.1	0.27749	6250	$x^{*}=(0.0003,0.9996,0.1028)^{T}$
			$y^{*}=(0.0006,4.9990,0.1030)^{T}$

**Table 3:** Numerical results of Example 6.1

Initial point: $x_{0}=(0.9528,0.7041,0.9539)^{T}$, $y_{0}=(0.5982,0.8407,0.4428)^{T}$			
Algorithm	Time (s)	No. Iterations	Approximate solution $(x^{*},y^{*})$
RACQA (1.8)	0.28871	6581	$x^{*}=(0.0107,0.9999,0.0130)^{T}$
			$y^{*}=(0.0240,5.0006,0.0131)^{T}$
Algorithm 4.1	0.27543	6004	$x^{*}=(0.0002,0.9996,0.1028)^{T}$
			$y^{*}=(0.0005,4.9990,0.1030)^{T}$
